# Spontaneous Bowel Perforation in a Neonate with Anorectal Malformation

**DOI:** 10.4103/1319-3767.74460

**Published:** 2011

**Authors:** Gursev Sandlas, Paras Kothari, Dinesh Sarda, Parag Karkera

**Affiliations:** Department of Pediatric Surgery, Lokmanya Tilak Muncipal General Hospital, Mumbai, India

**Keywords:** Anorectal malformation, perforative peritonitis

## Abstract

Gastrointestinal perforation in neonates with anorectal malformations is extremely uncommon. Delayed patient presentation is an important factor that demands special attention. We present a neonate with anorectal malformation and meconium peritonitis following spontaneous bowel perforation. A day 1 neonate was referred with features suggested of peritonitis. After adequate resuscitation and drainage under local anesthesia, patient was successfully operated for a sigmoid perforation and is now awaiting definitive surgery for the anorectal malformation.

## CASE REPORT

**Figure 1:**
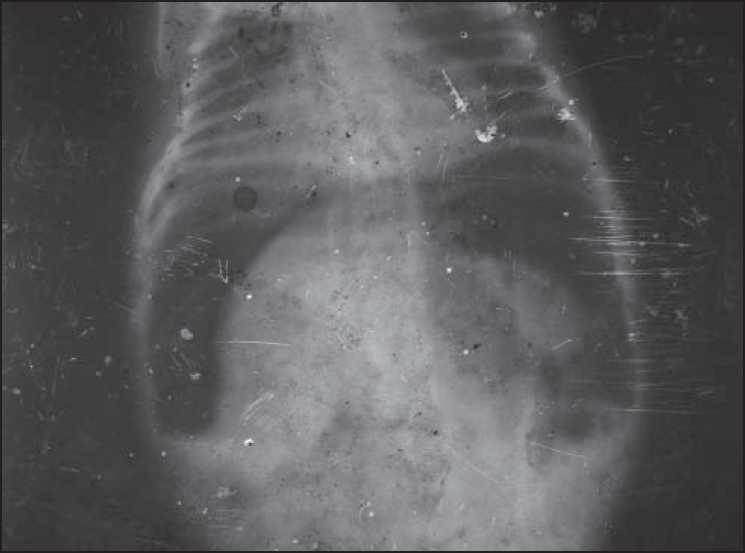
Abdominal X-ray showing large saddle-shaped shadow suggesting free peritoneal gas

A one-day old, full-term completed neonate, weighing 1.9 kgs, was admitted with abdominal distension and absent anal opening. There was no history of meconiumuria. On examination, the neonate was dehydrated, with hugely distended, soft, silent abdomen. There was no edema or erythema of the abdominal wall. Perianal inspection revealed absent anal opening with well-developed natal cleft and normal external genitalia. There was no evidence of meconium at the anal site or the penile tip. Abdominal X-ray suggested a large saddle-shaped air shadow below the diaphragm indicating free gas in peritoneal cavity. This was highly suggestive of a perforated viscous [Figure 1]. Hematological investigations were within normal limits apart from a low normal platelet count.

In view of poor general condition and abdominal distension causing respiratory distress, an abdominal drain was inserted under local anesthesia. Meconium and air were released, which decreased the abdominal distension. Adequate preoperative resuscitation (nasogastric aspiration, IV fluids, antibiotics, platelets and blood) was done and patient was taken up for exploratory laparotomy after an interval of about 24 h, which revealed features of meconium peritonitis. Rectum was ending above the levator ani complex suggesting a high variety of anorectal malformation (ARM). A longitudinal 5-mm perforation was found in the distal sigmoid colon. There was no evidence to suggest the concurrent presence of necrotising enterocolitis. The rest of the large bowel and small bowel was examined and found to be normal.

The perforation was closed in two layers; peritoneal lavage was given. As the sigmoid colon was the site of perforation, a right-sided loop transverse colostomy was done. The peritoneal cavity was closed in a single layer. The postoperative period was uneventful. Patient was discharged on post operative day 12. Patient is currently on regular follow up since last 3 months and doing well. Definitive surgery is planned at a later date.

## DISCUSSION

Gastrointestinal perforation is a rare clinical entity in neonates with ano-rectal malformations.[1–4] Lack of awareness makes early diagnosis and timely surgical intervention difficult, thereby resulting in high morbidity and mortality.

The etiopathogenesis of GI perforation in neonates with ARM is explained by a combination of factors.[1] The downstream occlusion results in proximal intestinal dilatation and increase in intraluminal pressure, resulting in tension gangrene.[1,4,5] In the presence of various factors such as delayed presentation and surgical intervention and absence of recto-urinary fistula, the gangrene progresses to GI perforation, with the large bowel being the most common site.[1] The non-viable intestine resulting from tension gangrene may undergo perforation even when the closed loop obstruction has been relieved, emphasizing the role of close clinical observation of such cases in the postoperative period.[1]

A high index of suspicion in neonates with ARM presenting with sepsis and features of neonatal peritonitis like tense, distended, silent abdomen with parietal wall edema and erythema provide a clue to the diagnosis.[1] Although features of pneumoperitoneum on abdominal X-ray have been reported in 60%-70% of neonates with GI perforation, its presence is confirmatory.[1,4,5] Contrast studies may sometimes be hazardous and are not routinely recommended in such sick neonates.[1,5] The management of GI perforation in neonates with ARM aims at aggressive resuscitation and early surgical intervention.[1] Preoperative stabilization improves the survival rate in such high-risk cases.[1,4,5] As experienced in the present case, preoperative drainage of intraperitoneal air by drain laparostomy is useful, especially in unstable neonates or those with severe respiratory distress.[1] The type of surgical intervention depends upon the physiological status of the patient, the site of perforation, the type of anorectal anomaly, and the degree of peritoneal contamination.[1] Although primary closure of perforation may be attempted in selected cases, exteriorization of the perforation as stoma or its primary closure with a proximal diverting stoma remains the treatment of choice.[1,4,5]
